# Socio-Demographic Factors Associated With COVID-19 Vaccine Hesitancy Among Middle-Aged Adults During the Quebec's Vaccination Campaign

**DOI:** 10.3389/fpubh.2022.756037

**Published:** 2022-03-18

**Authors:** Rodolphe Jantzen, Mathieu Maltais, Philippe Broët

**Affiliations:** ^1^CARTaGENE, Research Center, CHU Sainte-Justine, Montreal, QC, Canada; ^2^Université de Montréal, Montreal, QC, Canada; ^3^Faculté des Sciences de l'Activité Physique, Université de Sherbrooke, Sherbrooke, QC, Canada; ^4^University Paris-Saclay, CESP, INSERM, Villejuif, France; ^5^Assistance Publique-Hôpitaux de Paris (AP-HP), Hôpitaux Universitaires Paris-Sud, Hôpital Paul Brousse, Villejuif, France

**Keywords:** COVID-19, vaccine hesitancy, Quebec vaccination campaign, population-based cohort, CARTaGENE, tree-based model

## Abstract

**Introduction:**

The objective of this study was to characterize the combinations of demographic and socioeconomic characteristics associated to the unwillingness to receive the COVID-19 vaccines during the 2021 Quebec's vaccination campaign.

**Materials and Methods:**

In March-June 2021, we conducted an online survey of the participants of the CARTaGENE population-based cohort, composed of middle-aged and older adults. After comparing the vaccinated and unvaccinated participants, we investigated vaccine hesitancy among participants who were unvaccinated. For identifying homogeneous groups of individuals with respect to vaccine hesitancy, we used a machine learning approach based on a hybrid tree-based model.

**Results:**

Among the 6,105 participants of the vaccine cohort, 3,553 (58.2%) had at least one dose of COVID-19 vaccine. Among the 2,552 participants, 221 (8.7%) did not want to be vaccinated (91) or were uncertain (130). The median age for the unvaccinated participants was 59.3 years [IQR 54.7–63.9]. The optimal hybrid tree-based model identified seven groups. Individuals having a household income lower than $100,000 and being born outside of Canada had the highest rate of vaccine hesitancy (28% [95% CI 19.8–36.3]). For those born in Canada, the vaccine hesitancy rate among the individuals who have a household income below $50,000 before the pandemic or are Non-retired was of 12.1% [95% CI 8.7–15.5] and 10.6% [95% CI 7.6–13.7], respectively. For the participants with a high household income before the pandemic (more than $100,000) and a low level of education, those who experienced a loss of income during the pandemic had a high level of hesitancy (19.2% [8.5–29.9]) whereas others who did not experience a loss of income had a lower level of hesitancy (6.0% [2.8–9.2]). For the other groups, the level of hesitancy was low of around 3% (3.2% [95% CI 1.9–4.4] and 3.4% [95% CI 1.5–5.2]).

**Discussion:**

Public health initiatives to tackle vaccine hesitancy should take into account these socio-economic determinants and deliver personalized messages toward people having socio-economic difficulties and/or being part of socio-cultural minorities.

## Introduction

Less than 1 year after the WHO had declared the COVID-19 outbreak as a public health emergency of international concern, major pharmaceutical companies announced that vaccine candidates achieved great success in phase 3 clinical trials, paving the way to vaccination campaigns. In the autumn of 2020, each provincial governments in Canada began to plan the vaccination campaign for their residents while the federal department Health Canada conducted an independent scientific review of the candidate vaccines. Between December 2020 and March 2021, Health Canada approved four vaccines: two messenger RNA (Moderna, Pfizer-BioNTech) and two viral-vector-based vaccines (Janssen, AstraZeneca/COVISHIELD) (1).

In November 2020, the Quebec's immunization committee (Comité sur l'immunisation du Québec), a branch of the INSPQ (Institut national de santé publique du Québec), provided to the Quebec government a priority list with ten categories, which laid out the order in which Quebecers would be vaccinated (2). Age was the main factor of this priority list. The COVID-19 vaccination began in Quebec on December 14th. By mid-July, 72.4% of the Quebec population has received at least one dose of a COVID-19 vaccine with, as expected, a coverage among older adults higher than among young adults [96.3% (over 80 years) to 69.7% (18–29 years)] reflecting vaccine priority levels (3). At the same time, across Canada, 69.7% of the population has received at least one dose of a COVID-19 vaccine (4).

Even though Canada quickly moved to the top of the high-income countries for 1st doses of COVID-19 vaccine, the emergence of new more transmissible variants like B.1.617.2 (i.e., Delta variant) complicates the fight to subdue the pandemic, pushing to reach population immunity as soon as possible (5). However, vaccine hesitancy may jeopardize efforts to achieve this latter task. Vaccine hesitancy, as defined by the WHO SAGE Working Group on Vaccine Hesitancy, refers “to delay in acceptance or refusal of vaccination despite availability of vaccination services.” (6). As it is currently not mandatory to be vaccinated against COVID-19 in Quebec, one of the main goals for the public health authorities in the coming months is to understand the factors contributing to vaccine hesitancy.

In late 2020–early 2021, surveys have been launched in various countries for identifying factors associated with COVID-19 vaccine hesitancy (7–13). Most of these surveys have been conducted before the start of the vaccination campaigns when vaccines were not available and across countries with different health care systems, but as emphasized by the WHO SAGE Working Group, “vaccine hesitancy is complex and context specific, varying across time, place and vaccines” (6). Thus, few months after the start of the vaccination campaign in Quebec and with large supply of vaccines, the objective is now to better characterize the factors associated with the vaccine hesitancy among the unvaccinated population. This may help public health authorities to deliver more personalized messages about the benefits of vaccination.

In this context, the population-based cohort CARTaGENE, composed of middle-aged and older adults, which was established before the COVID-19 outbreak and is followed-up regularly, offered a unique opportunity to investigate the Quebec's COVID-19 vaccination campaign and to characterize the combinations of demographic and socioeconomic characteristics associated to the unwillingness to receive the COVID-19 vaccines.

## Materials and Methods

### CARTaGENE Population-Based Cohort

CARTaGENE is a population-based cohort composed of more than 43,000 Quebec residents aged between 40 and 69 years at recruitment (14). The original survey design was defined by gender, age groups and forward sortation area (defined by the first 3-digit postal codes). Participants were randomly selected to be broadly representative of the population recorded on the Quebec administrative health insurance registries [about 98% of Quebec residents (15)]. Participants have been recruited during two phases, in 2009–2010 (*n* = 23,000) and 2013–2014 (*n* = 20,000), in metropolitan areas where nearly 70% of Quebecers live. CARTaGENE is part of the Canadian Partnership for Tomorrow's Health (CanPath, former called CPTP) which is the Canada's largest population health research platform (16). More information can be found in the Supplementary Methods.

### CARTaGENE COVID-19 Online Questionnaire

For this study, we used the CARTaGENE COVID-19 follow-up online questionnaire, which is an extension of the COVID-19 questionnaire that has been sent in June–July 2020 during the spring outbreak (17). This online questionnaire was developed in close collaboration with the CanPath consortium (16). As for the previous questionnaire, a web-link to the consent and questionnaire was sent by email to the CARTaGENE participants with a valid email address (*n* = 33,019) between the end of March 2021 and May 2021. When the survey closed in early June 2021, 6,236 responded to the questionnaire.

### Outcomes and Selected Populations

**Figure 1:**
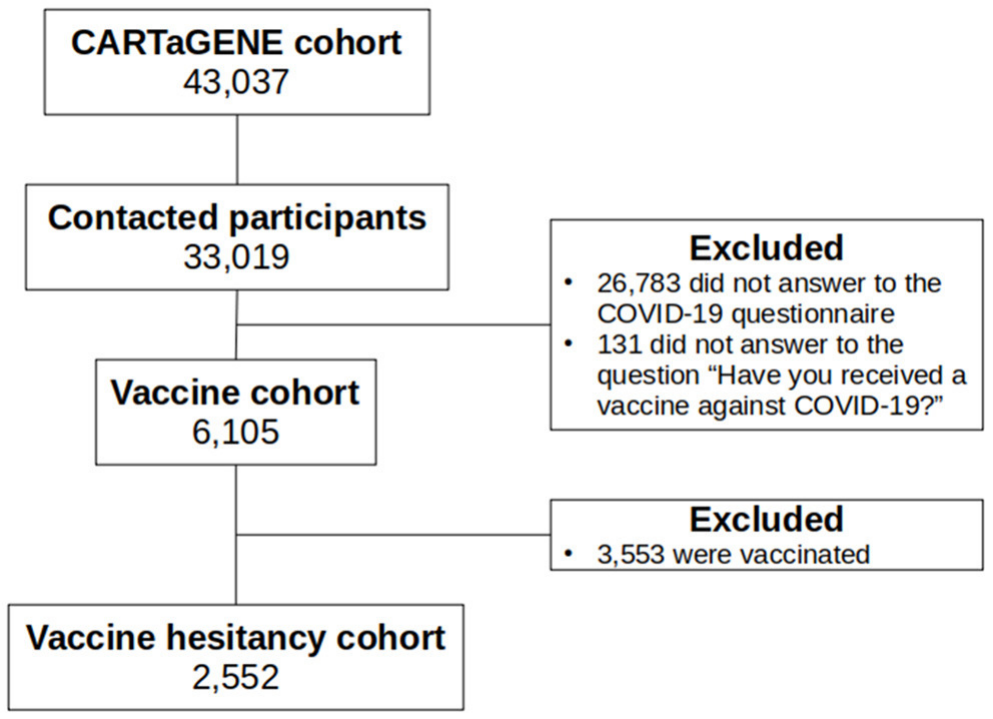
Flow-chart.

To describe the population under study and to compare vaccinated and unvaccinated participants, we selected participants that responded to the question “Have you received a vaccine against COVID-19?”, hereinafter called the “vaccine cohort” (Figure 1).

In this study, we used the term “vaccine hesitancy” to describe individuals who either rejected the vaccination or were unsure whether they would be vaccinated. In practice, we used the question “Would you be willing to take a vaccine against COVID-19?” (yes/no/don't know).

In the following, after comparing the characteristics of the vaccinated and unvaccinated individuals, we investigated vaccine hesitancy among participants who were unvaccinated (hereinafter called the “vaccine hesitancy cohort”, see Figure 1).

### Statistical Methods

#### Investigated Variables

We investigated different socio-demographic variables: age, gender, history of declared COVID-19 infection, country birth, current living region, highest level of education, being an essential worker, dwelling, annual household income before the pandemic, monthly household income changed and savings changed because of the COVID-19 pandemic, the impact of COVID-19 on the ability to meet financial obligations or essential needs, ethnicity, current employment status, Pre-existing medical conditions, current mental and emotional health (coded as continuous to allow for a more parsimonious regression model: poor = 1, fair = 2, good = 3, very good = 4, excellent = 5), and current mental and emotional health compared to before the pandemic. The education level was retrieved from the original CARTaGENE survey. The list of the variables and how they were encoded can be found in the Supplementary Methods.

#### Descriptive and Univariate Analyses

To assess the representativeness of the studied cohort, we compared the characteristics of the respondents to the Non-respondents and to the whole CARTaGENE cohort (age, gender, education level, country birth, and province birth).

To describe the characteristics of the populations, means with standard deviation (SD) and frequencies were reported for continuous and qualitative variables, respectively. The number of missing data per variables were also reported.

For comparing means between groups, we used *t*-test. For categorical variables, we used chi-squared test or exact Fisher test if needed. To take into account the multiple comparisons, *p*-values were corrected with the Benjamini and Hochberg method (18). We reported odds ratios (OR) with the 95% confidence interval (95% CI) only for variables with a *p*-value lower than 5%.

Finally, we plotted the number of newly vaccinated per day and compared the 50–69 and 70–79 age groups to the vaccination in Quebec (3).

#### Multivariate Analyses

In this work, we performed two multivariate analyses with two different objectives. For the comparison of vaccinated and unvaccinated individuals, our objective was to assess the relationship between the vaccination and socio-demographic factors whereas our main objective for the analysis of vaccine hesitancy was to identify homogeneous groups of individuals with respect to vaccine hesitancy. Moreover, and from a pragmatic point of view, the vaccine hesitancy analysis was performed among the unvaccinated individuals since identifying factors of hesitancy among this group may provide practical guidance for public health authorities.

For the first objective (comparison of vaccinated and unvaccinated), we performed a feature selection based on a penalized logistic regression approach (LASSO with the smoothing parameter chosen by cross-validation) to select the variables associated with the vaccination. Then, using the variables with Non-null regression coefficients, we fit a Non-regularized multivariate logistic regression model and reported the results (19).

For the second objective (vaccine hesitancy), since complex interplay between socio-demographic factors was expected, we considered a machine learning approach based on a hybrid tree-based model called Generalized Partially Linear Tree-based Regression model (GPLTR) (20). In this model, the linear part is used to adjust for the main effects of some confounder variables whereas the nonparametric tree part is used to capture the distributional shape of the data ensuing from the interplay of multiple explanatory factors. This approach provides a classification of individuals in homogeneous groups with respect to vaccine hesitancy and identifies relevant combination of explanatory variables taking into account confounding factors. In this study, since age was the main factor for vaccine priority, we adjusted for age effect in the linear part of the model. Using a penalized maximum likelihood method with the Bayesian information criterion (BIC), we selected the optimal hybrid tree-based model (20).

As regression-trees are known to be prone to instability which means that changes in the dataset may modify the structure of the tree, we also performed an ensemble analysis by fitting a series of hybrid tree-based models obtained from different bootstrap samples of the original dataset and reported the variable importance scores (21). Larger scores highlight which variables were most relevant for the vaccine hesitancy. In this work, this ensemble analysis was not intended to provide individual predictions for vaccine hesitancy but to give arguments regarding the reliability of the selected optimal tree-based model. In practice, we reported the deviance importance score (DIS) of each variable that was computed for each tree-based model as the sum of the values of the deviance at each split based on this variable (21). These scores were summed across the set of trees, and normalized to take values between 0 and 1, with the sum of all scores equal to 1. A set of 300 hybrid tree-based models was done and we reported the variables as ranked by the DIS.

These multivariate analyses were performed without replacing missing data.

Statistical analyses were performed using R software (22). Tree-based model analyses were performed with the “GPLTR” (Generalized Partially Linear Tree-Based Regression Model) R package (20).

## Results

### Vaccine Cohort Analysis

As compared to the Non-respondents (*n* = 26,783), the respondents (*n* = 6,236) were slightly older (63.8 vs. 62.2 mean years, *p* < 0.001), with more women (60.7 vs. 53.8%, *p* < 0.001), had a higher education level (graduate/university 59.4 vs. 45.5%, *p* < 0.001), were more frequently born in Canada (89.9 vs. 86.0%, *p* < 0.001) and in the Quebec province (85.1 vs. 80.1%, *p* < 0.001). The same results were observed when comparing the respondents to the whole CARTaGENE cohort.

**Figure 2:**
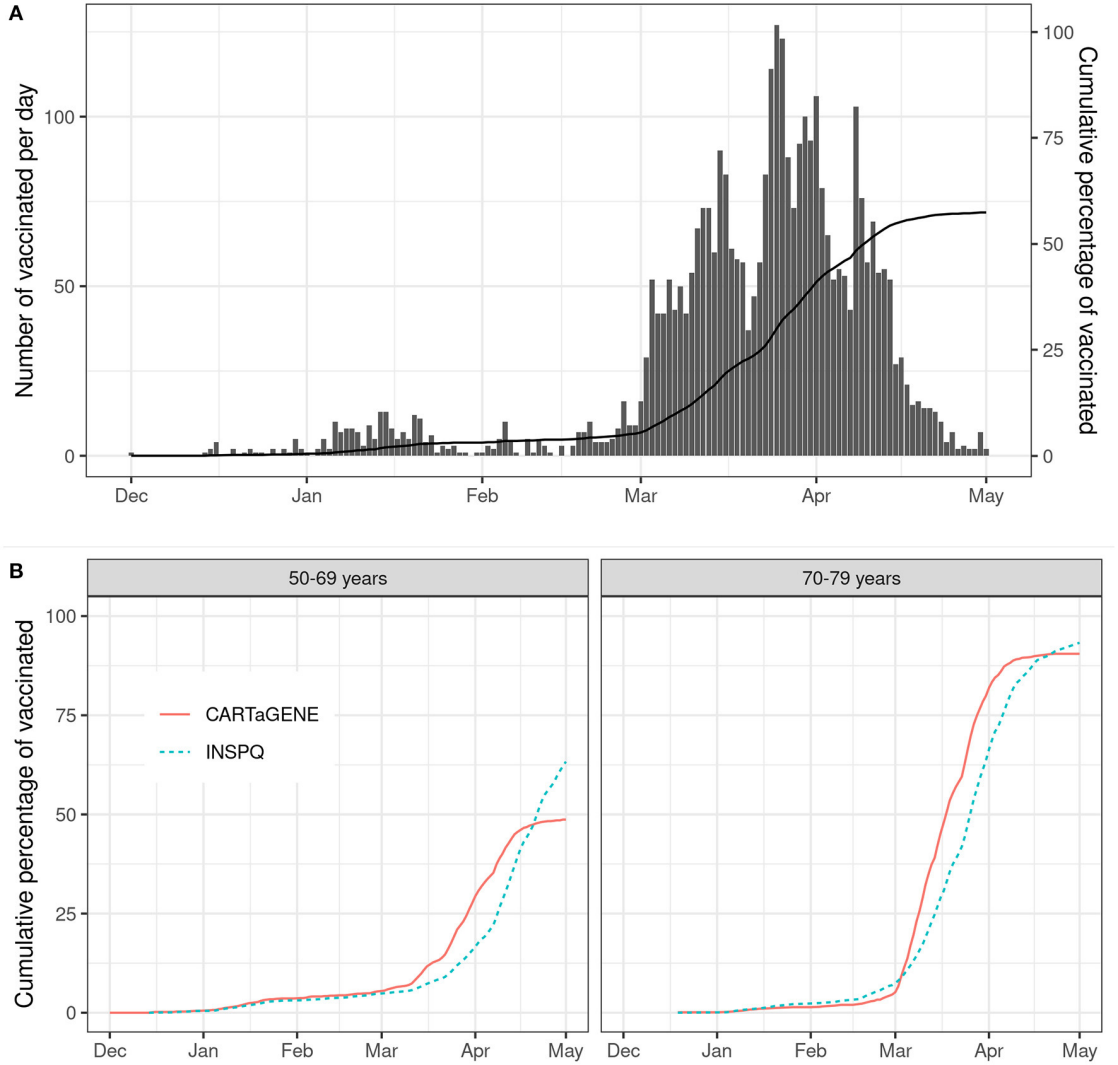
Evolution of vaccination from December 1st 2020 to May 1st 2021. **(A)** Number of vaccinated per day and the cumulative percentage of vaccinated in the CARTaGENE cohort; **(B)** Comparison of the cumulative percentage of vaccinated between the CARTaGENE cohort and the Quebec population [source: INSPQ (3)] in the 50–69 and 70–79 years groups. INSPQ, Institut national de santé publique du Québec.

Among the 6,105 participants of the vaccine cohort, 3,496 (57.3%) had one dose of COVID-19 vaccine, while only 57 (0.9%) had two doses. The mean age of vaccinated and unvaccinated was 66.8 years (SD 6.94) and 59.6 (SD 6.30), respectively (*p* < 0.001). The number of vaccinated per day increased substantially from March 2021 (Figure 2A), which was consistent with the number of vaccinated in the Quebec general population (Figure 2B). Pfizer was the most administered vaccine as the first dose (70.5%), followed by AstraZeneca (17.7%), Moderna (11.7%), Medicago (phase 3 clinical trial) (0.14%) and Janssen (0.03%). Characteristics of the vaccine cohort can be found in Supplementary Table S1.

Variables significantly associated with being vaccinated in univariate analysis are reported in Supplementary Table S2. In the multivariate logistic regression analysis performed with the factors selected by the penalized regression (Table 1 and LASSO coefficients in Supplementary Table S3), older participants and age-related factors (retirement home, being retired), having a Pre-existing medical condition and a good mental/emotional health were significantly associated with a higher rate of vaccination. A lower level of education, working full time/self-employed and living outside of Montreal were significantly associated with a lower rate of vaccination.

**Table 1:** Multiple logistic regression for being vaccinated.

	**OR [95% CI]**	***p*-value**
**Age (Ref:** **>60 years)**	3.78 [3.21–4.46]	<0.001
**Current living region (ref: Montreal)**	0.33 [0.28–0.38]	<0.001
**Dwelling**		
House	Ref.	
Apartment or condominium	1.08 [0.94–1.25]	0.3
Retirement home	8.97 [2.73–55.3]	0.003
Other	1.21 [0.40–3.83]	0.7
**Full time/self-employed**	0.75 [0.61–0.92]	0.007
**Pre-existing medical condition**	1.38 [1.20–1.58]	<0.001
**Highest level of education**		
0–Graduate studies/University	Ref.	
1–College	0.79 [0.69–0.91]	0.001
2–High school or less	0.83 [0.68–1.00]	0.052
**Ethnicity: Latin Hispanic**	0.49 [0.23–1.02]	0.059
**Current mental and emotional health (continuous: poor** **=** **1, fair** **=** **2, good** **=** **3, very good** **=** **4, excellent** **=** **5)**	1.09 [1.03–1.17]	0.005
**Impact of COVID-19 on the ability to meet financial obligations or essential needs**	0.83 [0.65–1.06]	0.14
**Retired**	1.46 [1.18–1.81]	<0.001

*CI, Confidence interval; OR, odds ratio; ref, reference*.

### Vaccination Hesitancy Analysis

Among the 2,552 unvaccinated participants, 221 (8.7%) did not want to be vaccinated (91) or were uncertain (130). The median age for the unvaccinated participants was 59.3 years [IQR 54.7–63.9], with 60.3% women. Characteristics of the vaccine hesitancy cohort can be found in Supplementary Table S4.

From univariate analyses, we found that participants born outside of Canada, with a lower level of education, a lower annual household income before the pandemic, a financial impact because of the pandemic, and from visible minority groups were more likely to be hesitant about COVID-19 vaccines. Participants older than 60 years, retired and that lived outside of Montreal were more willing to get a COVID-19 vaccine (Table 2).

**Table 2:** Variables significantly associated with vaccine hesitancy/refusal in univariate analysis.

	**OR [95% CI]**	***p*-value OR**	***p*-value overall**
**Age (ref:** ** <60 years)**	0.70 [0.53–0.93]	0.014	0.013
**Country birth (ref: Canada):**	3.21 [2.27–4.47]	<0.001	<0.001
**Current living region (ref: Montreal):**	0.42 [0.31–0.57]	<0.001	<0.001
**Highest level of education:**			<0.001
0 – < high school/High school	1.67 [1.08–2.52]	0.02	
1 – College	1.85 [1.36–2.50]	<0.001	
2 – Graduate studies/University	Ref.	Ref.	
**Annual household income before the pandemic:**			<0.001
0 – >$100,000	Ref.	Ref.	
1 – $50,000-$100,000	2.03 [1.41–2.95]	<0.001	
2 – < $50,000	3.47 [2.34–5.17]	<0.001	
**Monthly household income changed because of the COVID-19 pandemic:**			<0.001
0 – No change-Somewhat increased-Substantially increased	Ref.	Ref.	
1 – Substantially decreased-Somewhat decreased	1.94 [1.41–2.63]	<0.001	
**Savings changed because of the COVID-19 pandemic:**			<0.001
0 – No change-Somewhat increased-Substantially increased	Ref.	Ref.	
1 – Substantially decreased-Somewhat decreased	2.13 [1.53–2.93]	<0.001	
**Impact of COVID-19 on the ability to meet financial obligations or essential needs:**			<0.001
0 – No impact-Minor impact	Ref.	Ref.	
1 – Moderate impact-Major impact	2.72 [1.84–3.94]	<0.001	
**Ethnicity: White**	0.32 [0.22–0.48]	<0.001	<0.001
**Ethnicity: Black**	5.65 [1.91–14.9]	0.003	0.003
**Ethnicity: Latin Hispanic**	6.34 [2.76–13.8]	<0.001	<0.001
**Retired**	0.65 [0.48–0.88]	0.005	0.007

*CI, Confidence interval; OR, odds ratio; ref, reference*.

**Figure 3:**
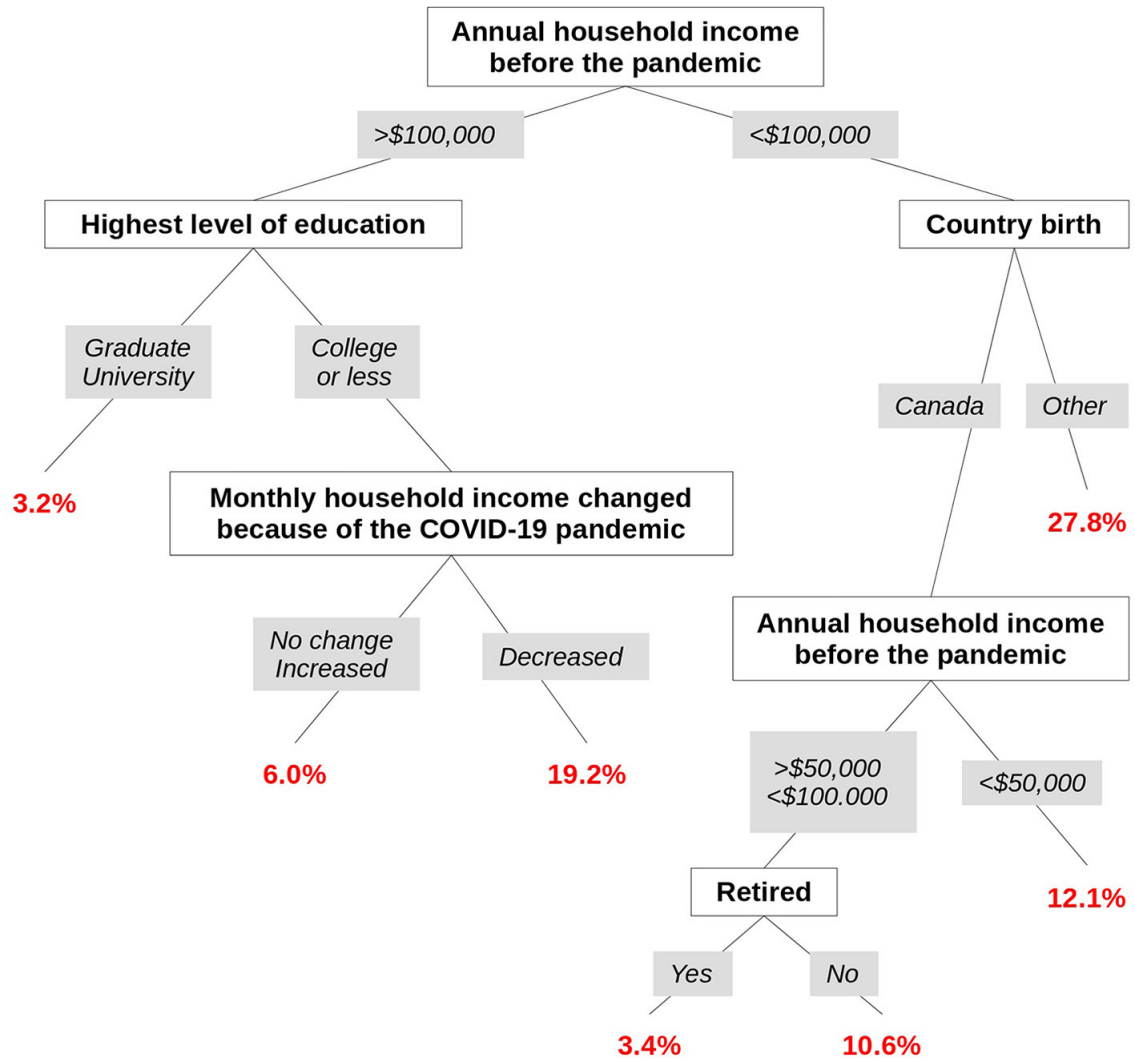
Tree-based multivariate model with adjustment on age for vaccine hesitancy.

Results from the tree-based multivariate analysis with adjustment on age identified various combinations of factors leading to homogeneous groups of individuals with respect to vaccine hesitancy. The optimal tree had seven leaves relying on five factors: household income before the pandemic, level of education, country of birth, impact of COVID-19 on the ability to meet financial obligations or essential needs, and being retired (Figure 3). The first split was based on the household income before the pandemic and split those having a high income (more than $100,000) from those having a low-intermediate household income. In this latter group, those being born outside of Canada had the highest rate of participants with vaccine hesitancy (32/83, 27.8% [95% CI 19.6–36.0]). For those born in Canada, the vaccine hesitancy rate was lower but still Non-negligible among those who had a low household income before the pandemic (below $50,000) or were active (Non-retired) people (43/312, 12.1% [95% CI 8.7–15.5] and 43/361, 10.6% [95% CI 7.6–13.7], respectively). In contrast, those who were retired had a low level of hesitancy (12/344, 3.4% [95% CI 1.5–5.2]).

For the participants with a high household income before the pandemic (more than $100,000) and a low level of education, those who experienced a loss of income during the pandemic had a high level of hesitancy (10/42, 19.2% [8.5–29.9]) whereas others who did not experience a loss of income had a lower level of hesitancy (13/203, 6.0% [2.8–9.2]). In contrast, for those with a high level of education, the level of hesitancy was low (23/706, 3.2% [95% CI 1.9–4.4]).

Table 3 displays odd-ratio estimates and *p*-values for age (confounding variable—linear part) and terminal leaves corresponding to the optimal hybrid tree-based model for being vaccine hesitant.

**Table 3:** Optimal hybrid tree-based model (GPLTR) analysis for vaccine hesitancy.

	**OR [95% CI]**	***p*-value**
**Age (Ref:** ** <60 years)**	0.70 [0.49–1.01]	0.056
**Groups (leaves of the tree)**		
Annual household income before the pandemic >$100,000 AND graduate/university level of education	Ref.	
Annual household income before the pandemic >$100,000 AND college or less level of education AND no decrease of monthly household income	1.97 [0.98–3.95]	0.058
Annual household income before the pandemic >$100,000 AND college or less level of education AND decrease of monthly household income	7.34 [3.28–16.44]	<0.001
Annual household income before the pandemic between $50,000 and $100,000 AND born in Canada AND retired	1.32 [0.63–2.76]	0.47
Annual household income before the pandemic between $50,000 and $100,000 AND born in Canada AND not retired	3.61 [2.14–6.08]	<0.001
Annual household income before the pandemic < $50,000 and AND born in Canada	4.83 [2.81–8.31]	<0.001
Annual household income before the pandemic < $100,000 AND born outside Canada	11.75 [6.56–21.05]	<0.001

*CI, Confidence interval; GPLTR, Generalized Partially Linear Tree-based Regression; OR, odds ratio; ref, reference*.

**Figure 4:**
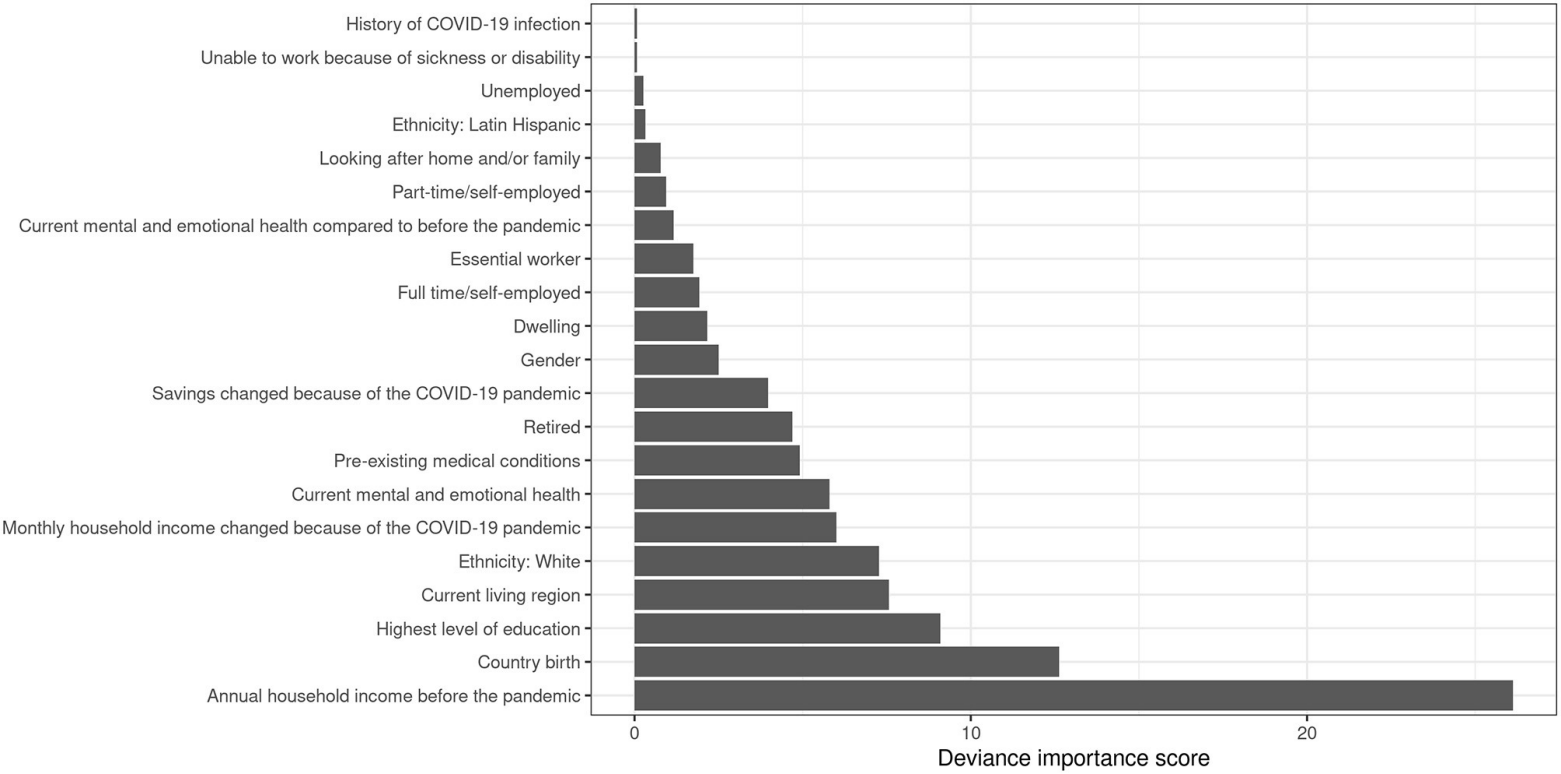
Normalized deviance importance score of the variables.

Results from the bagging procedure with a series of 300 bootstrapped hybrid tree-based models emphasized these results since the household income before the pandemic, the country of birth and the education level had the highest importance scores (Figure 4).

## Discussion

In a WHO report from 2019, vaccine hesitancy has been considered as one of the top ten global health threats (23). This issue clearly represents a challenge for the current COVID-19 pandemic control urging to conduct real-world studies in order to identify population groups that are most hesitant toward the COVID-19 vaccine.

In this cross-sectional study that was performed during the Quebec vaccination campaign, we showed several significant disparities between vaccinated and unvaccinated individuals, most of them being related to the Quebec vaccination plan. More precisely, the higher rates of vaccination for the older individuals, living in Montreal and in retired homes, and for those with Pre-existing health conditions can be linked to the Quebec multiple-phases vaccination plan (2, 24). Among the factors unrelated with the vaccination plan but already reported in previous studies, we have found that vaccination rates were correlated with the levels of education (8). We also reported that a good mental and emotional health was associated with a higher rate of vaccination.

When focusing on unvaccinated individuals, we have found that the global vaccine hesitancy rate in our cohort was low, as compared to other studies from other high-income countries (8.7 vs. 20–42% range) (7, 9–13, 25). However, it is worth noting that these studies were conducted when the COVID-19 vaccination campaign had not started. One study from Norway found a hesitancy rate close to our study in January 2021 (10.5%) (26).

In our study, some variables associated with vaccine hesitancy in univariate analysis were also found in the literature such as: older individuals, having a low level of education, a low income, being part of an ethnic minority group (7–13, 25, 27, 28). In contrast, other factors identified in previous studies were not found in our cohort like the gender or having a Pre-existing health condition (7–13, 25). However, it is worth noting that the definition of Pre-existing health conditions varies across studies. In this work, the Pre-existing medical conditions referred to pathologies defined as requiring a COVID-19 vaccine, according to the INSPQ (2).

Our multivariate tree-based analysis unraveled interesting combinations of socio-demographic factors regarding vaccine hesitancy. The highest rates of vaccine hesitancy were observed for the individuals born outside of Canada with a low/intermediate household income before the pandemic but also for those having a high household income before the pandemic but with a low level of education and who experienced a loss of income during the pandemic. Thus, even though the level of income before the pandemic is a major factor, there is no straightforward gradient between the level of income and vaccine hesitancy rates. Indeed, both the low and high income led to groups of individuals with either low or high hesitancy rates. This points out that vaccine hesitancy is rooted in more complex interactions of sociodemographic factors. These findings emphasize that vaccine hesitancy is linked to socio-economic issues that either Pre-existed before the pandemic or are due to the pandemic. Moreover, vaccine hesitancy seems also to be more severe for those belonging to socio-cultural minorities. Then, these findings strongly suggest that public health authorities should now deliver personalized messages to these groups using new channels (e.g., community leaders, charity, etc.).

The main strength of this cross-sectional study is that it relies on a well characterized population-based cohort. Thus, it offers a unique opportunity to characterize the factors associated with COVID-19 vaccination hesitancy during the spring 2021 vaccination campaign in Quebec. Moreover, the use of tree-based models provided a way to decipher various combinations of factors that interact each other that would have been overlooked by classical parametric regression approaches. As can be seen, the economic wealth status before the pandemic by itself is not sufficient to explain vaccine hesitancy but should be interpreted in the light of other factors such as the education and the socio-cultural background. Having in mind that tree-based models can be unstable, the results obtained from the bagging analysis confirmed the importance of the selected factors and strongly suggest that the final tree-based model is sufficiently reliable.

Our study has nevertheless some limitations. Firstly, our cohort is limited to middle and older adults that represents a group with high rates of vaccine willingness. However, in this age group, vaccine hesitancy may have the worst effect in terms of hospitalization and death. Also, despite this selection, we were able to identify combinations of factors that lead to high rates of vaccine hesitancy. Secondly, vaccine hesitancy was measured using only one item without investigating the perceived knowledge regarding vaccine risk/benefits. This objective was out of the scope of this study and would require another kind of study specifically focusing on the vaccine-hesitant individuals. Thirdly, as most studies, our series may have been affected by a potential selection bias, meaning that Non-respondents could have different opinions about vaccination compared to respondents. Fourthly and as previously discussed, vaccine hesitancy varies with time and place. Thus, any comparison with other studies should take into account that our study was done during the vaccination campaign in a high-income country with a publicly funded universal health care system. Nevertheless, these results highlight the importance of socio-economic and cultural factors in vaccine hesitancy that might be quite relevant in other countries. Fifthly, since the questionnaire was designed to cover a large spectrum of questions regarding the COVID-19 outbreak in Quebec, it did not include specific questions regarding defiance/mistrust toward authorities. In view of our results, further study focusing specifically on this topic would be worth to be done.

Finally, we think that the results of this study can be very useful for public health authorities in order to focus personalized messages toward more specific population subgroups.

## Conclusion

This study showed that specific combinations of socio-economic and cultural factors influence vaccine hesitancy among a population-based cohort composed of middle-aged and older adults from the Quebec province. Two groups have high rates of vaccine hesitancy. The first group is composed by individuals with a low/intermediate household income before the pandemic who are born outside of Canada. The second group is composed by individuals with a high household income before the pandemic but with a low level of education and who experienced a loss of income during the pandemic. In the coming months, public health initiatives to tackle vaccine hesitancy should take into account these determinants and deliver personalized messages toward these population groups.

## Data Availability Statement

The datasets for this article are not publicly available because the data were collected in the context of the CARTaGENE cohort. In accordance with the consent signed by the participants, data are accessible for research projects evaluated by Ethics Committees and by a committee on data and sample access. Requests to access the datasets should be directed to CARTaGENE (http://cartagene.qc.ca; access@cartagene.qc.ca; +1 514-345-2156).

## Ethics Statement

The studies involving human participants were reviewed and approved by the Research Ethics Board of the Sainte-Justine University Hospital Center (Number: FWA00021692). The patients/participants provided their written informed consent to participate in this study.

## Author Contributions

RJ: conceptualization, data curation, formal analysis, investigation, methodology, visualization, writing—original draft, and writing—review and editing. MM: resources and writing—review and editing. PB: conceptualization, formal analysis, methodology, project administration, supervision, validation, and writing—review and editing. All authors contributed to the article and approved the submitted version.

## Funding

We received from the Canadian Institutes of Health Research (CIHR) part of the funding allocated to the project SUrveilling Prospective Population cOhorts for COVID19 pRevalence and ouTcomes in Canada (SUPPORT-Canada), application ID: 447636. We received funding from the COVID-19 Immunity Task Force (CITF), application ID: 444098. The CIHR and CITF had no role in the design of the study and collection, analysis, and interpretation of data and in writing the manuscript.

## Conflict of Interest

The authors declare that the research was conducted in the absence of any commercial or financial relationships that could be construed as a potential conflict of interest.

## Publisher's Note

All claims expressed in this article are solely those of the authors and do not necessarily represent those of their affiliated organizations, or those of the publisher, the editors and the reviewers. Any product that may be evaluated in this article, or claim that may be made by its manufacturer, is not guaranteed or endorsed by the publisher.

## Supplementary Material

Supplementary Table S1 Vaccine cohort description.

Supplementary Table S2 Variables significantly associated with being vaccinated in univariate analysis.

Supplementary Table S3 Lasso coefficients.

Supplementary Table S4 Vaccine hesitancy cohort description.
