# Supplementary material for: Socio-Demographic Factors Associated With COVID-19 Vaccine Hesitancy Among Middle-Aged Adults During the Quebec's Vaccination Campaign
Source: Front Public Health. 2022 Mar 18;10:756037. doi: 10.3389/fpubh.2022.756037 (PMC8971569; doi:10.3389/fpubh.2022.756037)
Supplement: Supplementary file 1 [file Table_1.docx]

# CARTaGENE population-based cohort

With a rich collection of data including phenotyping and biological data, CaG is the largest ongoing prospective population cohort and biobank in Quebec, Canada.

The collected data includes a self-administered socio-demographic and lifestyle questionnaire (Phase A and B), an interviewer-administered health questionnaire (Phase A and B); non-invasive physical measurements (Phase A) and biospecimen collection (blood (Phase A and B) and urine (Phase A)). More than 30,000 individuals have provided blood samples.

The lifestyle questionnaires cover topics such as socio-demographic factors, lifestyle, mental state, psychosocial environment, personal and family history of disease, health care utilization, medication use, reproductive health and history and declared health conditions. A cohort-wide follow-up has been carried out in 2018 aiming at updating lifestyle and health information 5 to 10 years after the baseline data.

# Variables

- **Age** (continuous)
- **Gender** (male/female)
- **History of COVID-19 infection** (yes/no)
- **Country birth** (Canada/other)
- **Region of residence**: Montreal/other
- **Essential worker** (yes/no)
- **Dwelling**: Apartment or condominium / Retirement home / House / Other
- **Annual household income before the pandemic** (<$50,000 / $50,000-$100,000 / >$100,000)
- **Monthly household income changed because of the COVID-19 pandemic**: Substantially decreased-Somewhat decreased / No change-Somewhat increased-Substantially increased
- **Savings changed because of the COVID-19 pandemic**: Substantially decreased-Somewhat decreased / No change-Somewhat increased-Substantially increased
- **Impact of COVID-19 on the ability to meet financial obligations or essential needs**: No impact-Minor impact / Moderate impact-Major impact
- **Highest level of education**: Less than high school-High school / College / Graduate studies-University
- **Ethnicity** (yes/no for each): Arab, Black, Latin Hispanic, White
- **Current employment status**: as more than one employment status could be selected, we used one binary variable for each employment status (full time/self-employed, part-time/self-employed, retired, looking after home and/or family, unable to work because of sickness or disability, unemployed, unpaid or voluntary work student)
- **Current mental and emotional health in general (continuous)**: poor/fair/good/very good/excellent
- **Current mental and emotional health compared to before the pandemic**: worse/about the same-better
- **Pre-existing medical condition (yes/no):**
  - Diabetes
  - Cancer
  - High blood pressure (hypertension, not including during pregnancy), Heart attack (myocardial infarction), Heart failure Atherosclerosis / Coronary heart disease (including angioplasty or stents), Atrial fibrillation, Angina, Valvular heart disease (e.g. aortic stenosis, mitral valve prolapse)
  - Asthma, Chronic obstructive pulmonary disease (COPD), Interstitial lung disease, Chronic bronchitis, Cystic fibrosis, Emphysema, Sleep apnea
  - Crohn’s disease, Ulcerative colitis, Celiac disease
  - Liver cirrhosis, Chronic hepatitis, Fatty liver (NAFLD- non-alcoholic fatty liver disease / NASH – nonalcoholic steatohepatitis)
  - Acute renal failure, Chronic renal failure
  - Major depression, Minor depression, Bipolar disorder, Post-traumatic stress disorder, Schizophrenia or Schizoaffective disorder, Obsessive compulsive disorder, Anxiety disorder, Eating disorder, Addiction disorder (e.g., alcohol, drug or gambling dependence)
  - Thrombotic stroke, Hemorrhagic stroke, Multiple sclerosis
  - Rheumatoid arthritis, Osteoarthritis, Other type of arthritis
  - Lupus
  - Scleroderma
  - HIV, A weakened or compromised immune system such as Severe Combined Immunodeficiency, Hashimoto's thyroiditis, Sjögren’s syndrome, or Ankylosing spondylitis
